# Inference of stature using segmental measures in comparison with directly measured height in children and adolescents: an analytical cross-sectional study

**DOI:** 10.1016/j.jped.2024.05.004

**Published:** 2024-07-02

**Authors:** Isabela Lima Negrão, Márcia Reimol de Andrade, José Carlos R. Rodrigues, Yasmim de França S. Serapião, Laila Cristina M. Damázio, Joel Alves Lamounier, Jacqueline Domingues Tibúrcio

**Affiliations:** Universidade Federal de São João del-Rei (UFSJ), Departamento de Medicina, Campus Dom Bosco Campus, São João del-Rei, MG, Brazil

**Keywords:** Anthropometry, Stature, Body height

## Abstract

**Objectives:**

In the clinical routine of pediatricians, height is the most reliable indicator for assessing growth. However, there are situations where it is not possible to measure this parameter directly, making the estimation of height or length a useful alternative. The main goal of this study is to identify which segmental measure, including upper arm length (UAL), tibial length (TL), and knee-heel length (KHL), provides the stature estimate that most closely approximates directly measured height in the study participants**.**

**Methods:**

Analytical cross-sectional study of the anthropometric and segmental measures of 248 participants, aged 0 to 14 years old, using Stevenson's and Kihara's equations to estimate indirectly measured height.

**Results:**

The segmental measure that provided a measurement that deviated the least from the actual height was the KHL, followed by TL, both calculated using Stevenson's equations.

**Conclusion:**

The use of segmental measures to infer a child's stature is valuable in clinical practice, particularly in bedridden and incapacitated patients. Based on the present findings, the KHL and TL segments yielded more accurate results than the UAL

## Introduction

Anthropometry is a part of the routine clinical practice of pediatricians, who are required to regularly measure the weight, height, body mass index (BMI), and cephalic perimeter of patients. Among these parameters, height is the most accurate indicator for assessing growth,^1^^,^^2^ being also necessary for estimating relevant indices and markers in the field of health, such as BMI and body surface area (BSA); it is also important in determining the correct medication dosage and interpreting arterial blood pressure readings. However, there are situations where it is not possible to directly measure stature, such as in children or adolescents with neurological and/or muscular dysfunction, joint contractures, and spinal or thoracic deformities.^3^ In this context, alternative methods, such as body height estimation from segmental measures like upper arm length (UAL), tibial length (TL), and knee-heel length (KHL), can be useful in medical practice.^4^

Children with cerebral palsy (CP) face certain limitations when measuring stature and weight.^5^ The inability to maintain an orthostatic position, joint muscle contractures, muscle atrophy, the presence of scoliosis, and spasticity comprise limiting factors that hinder the measurement of height and weight using direct methods.^6^

An alternative for obtaining height data in children with CP is the application of equations to estimate actual height based on body segmental measurements. The equations described by Stevenson (1995)^5^ are commonly used in clinical practice and rely on linear proportional relationships between an individual's height and the measurements of UAL, KHL, and TL. However, these equations are more suitable for children with mild to moderate CP.^7^ Other equations have been proposed in the literature, such as those by Chumlea, Guo, and Steinbaugh (1994), which use the KHL as a predictive factor;^8^ Gauld et al. (2007), which are based on ulnar length;^9^ and the equations proposed by Kihara et al. (2015), which are based on TL.^10^ It is important to highlight that when estimating height through equations that use segmental measures, the generated errors may lead to misdiagnoses and inappropriate approaches regarding the child's nutritional status.

The aim of the present study was to determine which segmental measure, including UAL, KHL, and TL, provides the estimate that most closely approximates directly measured height in children. There is a need to establish more suitable associations between actual and estimated height in children, given the potential differences that may arise when comparing the calculated results based on the three segmental measurements with directly measured height.

## Methods

This analytical cross-sectional study was conducted with a real-life convenience sample of 248 children, aged 0 to 14 years old, who received care at the Pediatric Outpatient Clinic of the Maternal-Child Primary Health Unit in São João del-Rei, Minas Gerais, Brazil. Data were collected from March to October 2022 and included anthropometric measurements of weight and height/stature, as well as segmental measures (KHL, TL, and UAL). All patients who attended medical consultations in that period were included in the study. The parents and/or legal guardians signed an informed consent form (TCLE) for their child's participation in the study, and the children themselves signed an informed assent form (TALE). Patients with edema, amputations, muscular dysfunction, joint contractures, and spinal or thoracic deformities were excluded, as these factors made it impossible to directly measure stature.

The convenience sample was obtained from weekly pediatric outpatient consultations at the Health Center. Based on previous consultation records, a sample of 250 children was estimated, which is equivalent to 5% of the number of children attended per year.

To measure height, length measurements were taken in children under 2 years of age, in a supine position. In individuals over 2 years of age, on the other hand, stature was measured in orthostatic position, with their upper limbs positioned parallel to their body and their eyes looking straight forward. Furthermore, the children were asked to be barefoot or wearing socks, without the presence of hats, caps, or accessories. The instruments used in this assessment were the Center's scale anthropometer and an infantometer.

The UAL, TL, and KHL measurements were obtained using a FitMetria inextensible measuring tape, with a maximum length of 2 m (m) and precision of 0.05 cm (cm). To measure the UAL, the child was placed in an orthostatic position, with one arm flexed and with the palm facing upward. The measurement was performed on the non-dominant arm, at the midpoint between the acromion and the olecranon. Regarding the KHL, the child was placed in a sitting position, with their knees and heels flexed at the right angle. The measuring tape was placed on the heel of the non-dominant foot, over the fibular head. As for the TL, it was determined by measuring the length from the medial tibial condyle to the edge of the medial malleolus, with the patient in an orthostatic position and their feet spread hip-width apart.

All measurements were taken in duplicate. In cases where the divergence was greater than 2 mm (mm), a third measurement was taken, and the final value considered was the simple arithmetic average between the two closest measurements.

This study was approved by the Ethics Committee on Research Involving Human Beings - São João del-Rei Educational Units (CEPSJ), under CAAE No. 53879321.0.000005151.

The data were organized in an Excel 2016 spreadsheet containing the following variables: age, sex, KHL, TL, UAL, and directly measured height.

Stevenson's and Kihara's equations were used to estimate stature using the segmental measures. For upper arm length (EQ1), it was used Stevenson's Equation S = (4.35 X UAL) + 21.8; for knee-heel length (EQ2), S = (2.69 X KHL) + 24.2; for tibial length (EQ3), S = (3.26 X TL) + 30.8; and, for tibial length for typical development (EQ4), it was used the Kihara's Equation S = TL X 3.25 + 34.45. It is noteworthy that none of the children in the sample failed to have their anthropometric measurements taken; therefore, there were no missing data.

For the descriptive analysis of the data, the mean, median, standard deviation, coefficient of variation (CV), and the absolute (n) and relative (%) frequencies were considered. Statistical inferences were made using the Friedman and Wilcoxon tests for comparisons (dependent data) of the measured height and the estimated heights using Stevenson's and Kihara's equations. The Mann-Whitney test was used when comparing gender-based estimates (independent data). Statistical significance was considered at a p-value < 0.05, and the analysis was performed using the Minitab software, version 18.

## Results

A total of 248 children were assessed, of whom 116 (46.8%) were female and 132 (53.2%) were male. The study participants were distributed by age group: (1) from 0 to 28 days, the absolute frequency (n) was 10 and the relative frequency was 4.0%; (2) from 29 days to 1 year, 11 months, and 29 days, the absolute frequency (n) was 110 and the relative frequency was 44.4%; (3) from 2 years to 5 years, 11 months, and 29 days, the absolute frequency (n) was 69 and the relative frequency was 27.8%; (4) from 6 years to 9 years, 11 months, and 29 days, the absolute frequency (n) was 38 and the relative frequency was 15.3%; (5) and, from 10 to 14 years the absolute frequency (n) was 21 and the relative frequency was 8.5%.

**Figure 1:**
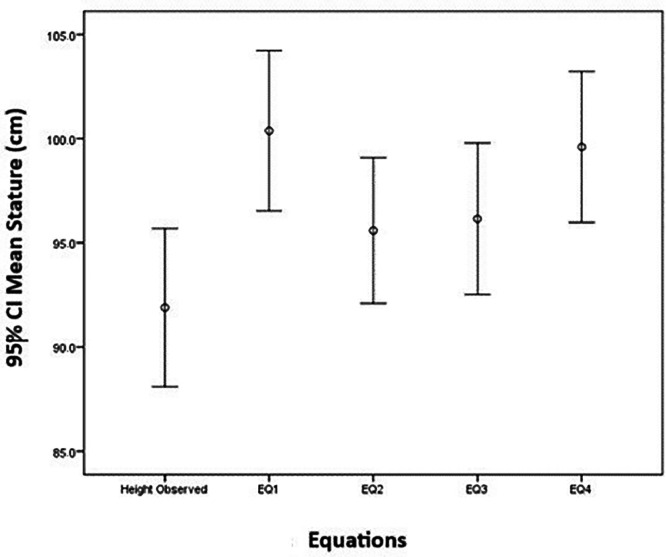
Confidence interval of 95% for the means of the directly measured stature and of the segmental measures (UAL - TL - KHL). UAL = upper arm length. TL = tibial length. KHL = knee-heel length.

The data showed that the mean height of the children was 91.9 cm, with a standard deviation of 30.3 cm, a median height of 87.7 cm, and an interquartile range of 51,0 cm. The mean height estimated by EQ1 (UAL) was 100.4 cm, with a standard deviation of 30.7 cm, a median of 94.4 cm and an interquatile range of 49.8 cm. The mean height estimated by EQ2 (KHL) was 95.6 cm, with a standard deviation of 30.0 cm, a median of 91.5 cm and an interquatiles range of 48.4 cm. Meanwhile, the mean height estimated by EQ3 (TL) was 96.1 cm, with a standard deviation of 29.0 cm, a median of 89.5 cm and an interquatile range of 52.2 cm. The estimates derived from EQ4 (Kihara) presented a mean of 99.6 cm, with a standard deviation of 28.9 cm, a median of 92.9 cm and an interquatile range of 52.0 cm. The estimates for measured height and estimated height using Stevenson's and Kihara's equations showed moderate homogeneity (CV of approximately 0.30) and close approximation when compared to each other. Figure 1 shows the 95% confidence intervals for the estimated mean height.

**Figure 2:**
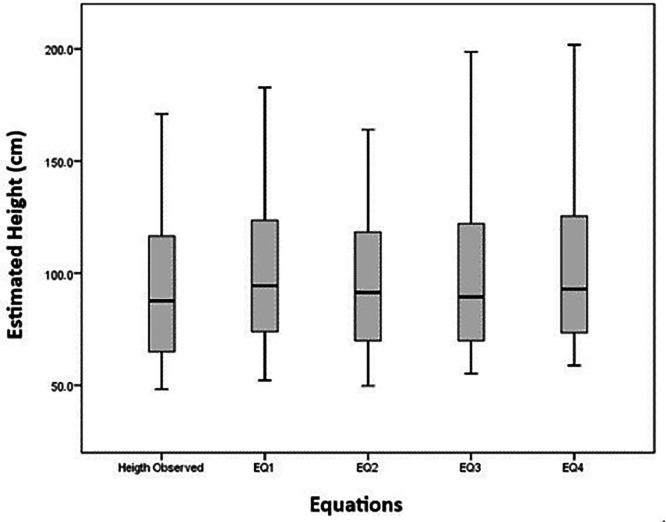
Boxplot graph of the means of the directly measured height and the segmental measures (UAL - TL - KHL). UAL = upper arm length. TL = tibial length. KHL = knee-heel length.

Figure 2 shows that the height estimates were not symmetrically distributed around the mean (p-value < 0.05). With 95% confidence, there was evidence of a statistically significant difference between the medians of the measured height and the estimated heights (p-value < 0.001), as determined by the Friedman test.

Comparisons between the medians of the measured height and the heights estimated by the equations were analyzed using the Wilcoxon test, and all of them were significant (Table 1). The smallest difference between medians was associated with EQ3.

**Table 1:** Differences between the medians of measured height and of the heights estimated by the equations.

Measurements	Difference between medians	*p*-value
Height (cm) - EQ1	−6.7	0.0001
Height (cm) - EQ2	−3.8	0.0001
Height (cm) - EQ3	−1.8	0.0001
Height (cm) - EQ4	−5.2	0.0001

Compared to the medians of the heights estimated using Stevenson's and Kihara's equations, the medians of the heights estimated by the equations EQ2-EQ3 (*p* = 0.255) and EQ1- EQ4 (*p* = 0.985) were not significant, whereas the others were significant (Table 2). In this case, the smallest differences were associated with EQ4.

**Table 2:** Differences between the medians of the heights estimated using the equations.

Measurements	Difference between medians	*p*-value
EQ2-EQ1	−2.9	0.0001
EQ3-EQ1	−4.9	0.0001
EQ4-EQ1	−1.5	0.0001
EQ4-EQ2	1.4	0.0001
EQ4-EQ3	3.4	0.0001

## Discussion

Calculating stature using segmental body measurements in children or adolescents is of utmost clinical importance because it helps predict height in individuals with functional or neurological limitations for traditional methods. The present study identified that the measurements of KHL and TL allowed for the calculation of indirectly measured height using Stevenson's equations.

The longitudinal bone growth of the lower limbs is much greater than that of the upper limbs, thus allowing for more accurate measurements.^11^ Similar results to those found herein were observed in the studies by Amezquita and Hodgson (2014) and Teixeira and Gomes (2014).^12^^,^^13^ The first study indirectly measured stature using the KHL segment with Stevenson's equation (1995),^5^ but only 40% of the sample had directly measured stature compared to the indirectly measured stature using KHL.^12^ In the second study,^13^ the stature calculated using the KHL was observed in children up to 3 years old. In the present study, all children and adolescents had their heights directly measured for comparison, and the age group was broader, between 0 and 14 years of age. It is noteworthy that for the KHL, Stevenson's equation (EQ3) demonstrated more accuracy when compared to both of Kihara's equations (EQ4 and EQ5).

Anthropometry is a powerful tool in clinical practice to evaluate the longitudinal growth and nutritional status of children.^1^^,^^2^ The systematic measurement of anthropometric data combined with the monitoring of development throughout childhood and adolescence can be a predictor of health.^14^ Measuring length and height allows for monitoring longitudinal growth and provides the foundation for calculating the Body Mass Index (BMI), which is crucial in classifying an individual's nutritional status based on WHO growth charts (2006).^15^ With the alarming rise in childhood and adolescent obesity,^16^^,^^17^ precision in performing anthropometric measurements has become essential. However, difficulties may arise in obtaining direct length and height measurements in individuals with motor limitations, such as cerebral palsy. In such cases, it is possible to use segmental measurements of upper and lower limbs to calculate length or stature through mathematical calculations.^5^^,^^7^^,^^9^^,^^10^^,^^12^

In the study by Lamounier et al. (2020),^6^ the authors stated that different results can be observed in the measurement of stature in the same individual with spastic cerebral palsy and lower-limb hypoplasia depending on the mathematical formula used to estimate stature indirectly. In this case, there may be a measurement bias in the calculation of stature using KHL and TL, as observed in the studies by Haapala et al. (2015)^18^ and Hogan (1999).^19^

The main contribution of the present study is the possibility of calculating indirectly measured stature using segmental measures in children and adolescents whose directly measured height is already known. This way, it is possible to determine which segment provides an indirect measurement of stature that is closest to the individual's actual height. This methodology can be replicated in other settings, making it feasible for other studies. Consequently, this study demonstrated that the mean values and standard errors of the segmental measurements and the directly measured KHL and TL provided results that were closer to the individual's actual height, i.e., the directly measured height. This result can be explained by the greater longitudinal bone growth of the lower limbs compared to that of the upper limbs, allowing for more accurate measurements.

The convenience sample, in a real-life scenario, revealed another fact that was not the initial objective of this study. The research was conducted on children and adolescents seeking healthcare at the primary health unit during a period that spanned from autumn to the beginning of spring, thus encompassing the entire winter season. The majority of these patients fell within the age groups of infants and preschoolers. This result can be explained by the seasonality of respiratory diseases, which aligns with WHO data, indicating that these diseases most frequently affect children under age 5, including a higher prevalence of pneumonia.^20^ This fact may explain why children under 5 years of age the largest contributors to this study were.

## Conclusion

The use of segmental measurements to infer a child's stature holds significant value in clinical practice, especially for bedridden and incapacitated patients. In this case, based on these findings, the KHL and TL segments provided better results than UAL in patients with known height, allowing for greater accuracy of these anthropometric measures. In perspective, for patients with motor limitations, such as cerebral palsy, the same methodology can be applied, using the sum of the segments as a model for directly measured height.

## Conflicts of interest

The authors declare no conflicts of interest
